# Overexpression of the aldehyde dehydrogenase *AhALDH3H1* from *Arachis hypogaea* in soybean increases saline-alkali stress tolerance

**DOI:** 10.3389/fpls.2023.1165384

**Published:** 2023-03-28

**Authors:** Yingxue Cao, Jing Wang, Siqi Zhao, Qingxi Fang, Jingwen Ruan, Shuanglin Li, Tongxin Liu, Yuxin Qi, Ling Zhang, Xiaoming Zhang, Fanli Meng

**Affiliations:** ^1^ Department of Agriculture, Northeast Agricultural University, Harbin, China; ^2^ Northeast Institute of Geography and Agroecology Chinese Academy of Sciences, Harbin, China; ^3^ Institute of Agricultural Biotechnology, Jilin Academy of Agricultural Sciences, Changchun, China; ^4^ Mudanjiang Branch of Heilongjiang Academy of Agricultural Sciences, Mudanjiang, China; ^5^ Heilongjiang Green Food Science Research Institute, Harbin, China

**Keywords:** aldehyde dehydrogenase, saline-alkali stress, transcriptome, metabolome, tolerance, soybean

## Abstract

Soybean production is severely hampered by saline-alkaline stress caused by saline-alkalization. Plants have aldehydrogenase (ALDH) family members that convert reactive aldehydes to carboxylic acids to remove active aldehyde molecules. However, little is known about the increased saline-alkali tolerance caused by the ALDH function in soybean. Here, we introduced a previously identified ALDH coding gene *AhALDH3H1* from *Arachis hypogaea* into the soybean genome to investigate its critical role in response to saline-alkali stress. Transgenic soybean with increased aldehyde dehydrogenase activity showed significant tolerance to saline-alkali stress. It reduced malondialdehyde (MDA) content compared to its receptor, suggesting that over-expression of *AhALDH3H1* accelerated soybean tolerance to saline-alkali stress by increasing aldehyde dehydrogenase activity, which is responsible for scavenging toxic MDA. To further analyze the inner mechanisms that allow transgenic plants to tolerate saline-alkali stress, we sequenced the transcriptome and metabolome of P3 (wild type, WT) and transgenic lines which were separately treated with water and a saline-alkali solution. When subjected to saline-alkali stress, the integrated analysis of the transcriptome and metabolome suggested that several genes related to cell wall structure crucial for preserving cell wall extensibility and plasticity were largely responsible for restoring homeostasis within the transgenic cells compared to WT. Metabolites, including both necessary ingredients for cell wall genesis and harmful production produced during the saline-alkali stress response, could be transported efficiently with the help of the ABC transporter, reducing the negative effects of saline-alkali stress. These findings suggest that introducing *AhALDH3H1* increases transgenic soybean tolerance to saline-alkali stress may through cell wall structure maintenance and metabolites transport.

## 1 Introduction

Plants, unlike animals, lack the ability to move independently due to their sessility. Therefore, they are unable to escape stresses, such as drought, salinity, heat, cold, pests, and pathogens. Hence, plants must evolve to respond and adapt to the surrounding environment. Among the various abiotic stresses, saline-alkali stress is an increasing stress that plants have faced in recent decades due to the growing human population and consequent food demands. Severe saline stress is always accompanied by high pH values rather than saline or alkali stress occurring independently. In addition, the synergistic effects of the two types of stresses combined will be more severe to normal growth and development of plants than the effect of either single stress alone (Fang et al., 2021).

The aldehyde dehydrogenases (ALDHs), a supergroup of enzymes responsible for removing active aldehyde molecules, convert various reactive aldehydes to their corresponding carboxylic acids with NAD+ or NADP+ as a cofactor to provide cells with potential protection against reactive oxygen species (ROS) (Brocker et al., 2013). A total of 14 distinct families of ALDH have been identified, with seven members including ALDH10, ALDH12, ALDH19, ALDH21, ALDH22, ALDH23, and ALDH24 identified only in plant species (Chen et al., 2002; Hou and Bartels, 2014). Previous studies have identified a series of ALDHs in various plant species, including *Arabidopsis thaliana* (Kirch et al., 2004)*, Chlamydomonas reinhardtii* (Brocker et al., 2013)*, Oryza sativa* (Gao and Han, 2009), *Solanum lycopersicum* (Jimenez-Lopez et al., 2016), and *Zea mays* (Jimenez-Lopez et al., 2010). A genome-wide strategy was recently used to identify 71 ALDHs in *Arachis hypogaea* (Zhang et al., 2023). ALDH family members play an essential role in mitigating abiotic stresses. In *Arabidopsis thaliana*, heat stress leads to an induced expression of ALDH3I1 and ALDH7B4 (Zhao et al., 2017). Compared with a single stress, the combination stresses of dehydration-heat, heat-salt, and wounding-heat cause an ALDH7B4 accumulation response. Compared to wild type plants, the *aldh3i1*, *aldh7b4* double mutant exhibited sensitivity to heat and stress combinations. Over-expression of *ALDH7B1-5A* from wheat enhanced the drought stress tolerance of *Arabidopsis thaliana*. By up-regulating stress responsive genes, water retention, contents of chlorophyll, and malondialdehyde (MDA) balance was maintained which enables transgenic plants to better tolerate drought stress (Zhao et al., 2017). MDA is an aldehyde that can directly interact with DNA and protein in plant species (Schmid-Siegert et al., 2012).

Soybean (*Glycine max(L.) Merr.*), a vital global crop, provides high-quality plant protein and oilseed production to humans and animals (Kou et al., 2022). Chinese soybean production-consumption occupies a relatively large global proportion compared to other countries because of the high rate of soybean consumption in China. This consumption rate increases the demand for soybeans. However, unavoidable stresses, especially saline-alkali stress, severely limit soybean production.

According to reported data, saline-alkali soil covers more than 950 million ha worldwide (An et al., 2016), and this figure is rapidly increasing yearly. The increasing saline-alkaline soil coverage reduces arable land utility, negatively impacting agricultural production (Bailey-Serres et al., 2019). Plant longevity is dependent on normal growth, development, and reproduction. Hence, the normal life of plants will be severely disrupted when exposed to a saline-alkali environment. However, previous research has mainly focused on salt stress and little is known about the mechanisms responsible for saline-alkali stress in soybean. Recently, we have identified a series of *AhALDH* members which are critical for saline-alkali stress response (Zhang et al., 2023). *Arachis hypogaea* and *Glycine Max* belong to the family of legumes, so we were curious whether these identified *AhALDH* genes have similar functions in soybean. To address this question, we introduced the *AhALDH3H1* gene into the soybean genome and evaluated its function in the saline-alkali stress tolerance process. Furthermore, we conducted transcriptome and metabolome sequencing to uncover the pathways and mechanisms responsible for increased tolerance of saline-alkali stress in soybean.

## 2 Materials and methods

### 2.1 Plant materials and growth conditions

The receptor soybean P3 used for transgenic plant creation was a previously obtained germplasm. All soybean plants used in this study were grown in the greenhouse of Northeast Agriculture University, Harbin, Heilongjiang province (126°E, 45.75°N) under a 16/8 dark photoperiod. The root tissue used for transcriptome and metabolome analysis was obtained from plants that had been treated with water and pH 8.9 saline-alkaline solution for 28d after 14d being planted in pots.

### 2.2 Constructs creation and transformation

The backbone vector for creating transgenic soybean is a modified *pCAMBIA1300* containing kanamycin and Spectinomycin as selection markers. First, we amplified the entire length CDS of *AhALDH3H1* from *Arachis hypogaea* cDNA using specific primers (**Table S1**). Next, the original vector and the amplified fragment were double-digested with KpnI and BamHI before ligating the CDS fragment and linearized the *pCAMBIA1300* vector with a T4 DNA ligase kit (Thermofisher Scientific, Cat#15224041). The ligation product was cultured overnight at 37°C after transformed into commercially competent cells. Overall, five positive colonies were selected and confirmed with a pair of specific primers. Finally, using correct constructs, Agrobacterium-mediated transformation was performed using *Agrobacterium tumefaciens* strain *GV3101* (Koncz and Schell, 1986). The construct of *proAhALDH3H1::GUS* was created by fusing the *AhALDH3H1* promoter which is a 2897bp DNA fragment with the β-glucuronidase(GUS) gene. Transformation was conducted by flower dipping method using Arabidopsis thaliana ecotype *Col-0*.

### 2.3 PCR and quantitative real-time PCR (qRT-PCR)

Total DNA was isolated using a DNA extraction kit (Kangwei Century Biotechnology Co., Ltd., Beijing, China). The PCR test was conducted with transgenic plant DNA or wild type (WT) DNA as a template, the specific primers listed in **Table S1**, distilled water, and KOD-One ™ PCR MasterMix (Lot# 153100) from TOYOBO. The detailed program was as follows: 98°C, 5 min; 98°C, 10 s; 58.8°C, 15 s; 68°C, 1 min; 68°C, 5 min; 12°C, maintenance. Agarose gel electrophoresis with approximately 1.8% agarose gel was used to test the PCR products.

qRT-PCR was also conducted here. The total RNA was isolated from both WT and transgenic lines using an OminiPlant RNA Kit (Lot# CW2598S) according to the manufacturer’s instructions. The cDNA was synthesized in a two-step strategy using HiScript II Q Select RT SuperMix for qPCR kit (Cat# R232-01) from Vazyme, and 1 µg isolated total RNA was used as a template. qRT-PCR was performed using a ROCHE LightCycler 96 machine and AceQ qPCR SYBR Green Master Mix (Cat# Q111-02) kit from Vazyme according to the manufacturer’s instructions. The qRT-PCR analysis results were evaluated using 2^-ΔΔ^CT (Livak and Schmittgen, 2001). Each sample was subjected to three independent biological replicates. *Actin 7* was used as an internal control. **Table S1** lists all the primer sets designed in this study.

### 2.4 Acetaldehyde dehydrogenase activity assay

Acetaldehyde dehydrogenase activity was determined using a MICHYBIO kit and a coupled enzyme assay in which acetaldehyde was oxidized by ALDH and produced NADH, which reacted with a probe to produce a colorimetric (450 nm) product proportional to the amount of ALDH activity present. The amount of enzyme which will generate 1 mole of NADH per minute at pH 8.0 and at a temperature of 25°C was determined using one unit of ALDH. Plant tissue (0.1 g) was ground in a prechilled motor with 1 ml isolation buffer. We further centrifuged the ice-chilled homogenate at 10000 g for 20 min at 4°C. The supernatant was collected and chilled before being measured. The Microplate Reader was preheated for 30 min, and the wavelength was set at 450 nm. The reaction mixes were set up according to the kit instructions, then thoroughly mixed by pipetting and the appropriate reaction mix was added to each well. After 4 min, the initial absorbance at 450 nm was measured as A1 and the final measurement absorbance as A2. Using the formula △A = A2-A1, we calculated the measurement change from initial to final for the samples. The standard curve can be calculated as y = 0.0021x-0.0044, R^2 =^ 0.9942, where x represents the standard control concentration (nmol/ml), and y represents △A. We calculated the ALDH activity as (nmol/min/g fresh weight) = (△A=0.0044)/0.0021*volume of the isolation buffer/weight/reaction time.

### 2.5 SOD activity and malondialdehyde content measurement

The superoxide dismutase (SOD) activity was evaluated using the corresponding kit from MIICHYBIO. WST-8 (4-[3-(2-methoxy-4-nitrophenyl)-2-(4-nitrophenyl)-2H-5-tetrazolio]-1,3-benzene disulfonate sodium salt) based method was used here as previously described (Beauchamp and Fridovich, 1971). MDA content was determined using a method that involved reacting MDA with thiobarbituric acid (TBA) following the instructions of the manufacturer.

### 2.6 Saline-alkaline treatment

The saline-alkali treatment was performed here. The saline-alkali solution with a pH value of 8.9 was prepared as follows: Every 1 L solution contained 2.925 g NaCl, 5.3 g Na_2_CO_3_, 37.8 g NaHCO_3,_ and 63.9 g Na_2_SO_4,_ in a 1: 1: 9: 9 ratio. The original solution was then diluted to 12.5 L and used for saline-alkali treatment. On Sep 1, 2022, we planted six pots of each transgenic line, including L1-1, L2-1, L3-1, L4-1, and WT in a mixture of soil and vermiculite at a 1:4 ratio for both saline-alkali and water treatments. Each pot contained 10 seeds and there were three biological replicates for each group. All plants were watered with tap water and unhealthy plants were removed over the next week; five healthy and identical individuals from each group were saved for further study. On Sep 14, 2022, two weeks after planting, the treatment group was watered with a saline-alkaline solution, in contrast to the control group treated with tap water. 28 d after treatment, the physiological index was tested and measured.

### 2.7 Metabolome and transcriptome analysis

Metware Biotechnology Inc. conducted the metabolome and transcriptome analysis. Twelve samples were classified into four groups, including the transgenic line and WT, which were subjected to either water or saline-alkaline stress (OE-W, OE-S, WT-W, and WT-S). Detailly, 2g fresh root tissues from WT and transgenic lines that both treated with water and saline-alkali stress for 28d were sampled. The total RNA was isolated from both WT and transgenic line using an OminiPlant RNA Kit (Lot# CW2598S) following the manufacturer’s instruction. Using Oligo(dT) tagged magnetic beads, mRNA was enriched and then sonicated randomly. The first strand of cDNA synthesized with random primers, followed by second strand of cDNA synthesis with buffer, dNTPs, RNase H and DNA polymerase I mixture. Then, double strands cDNA was purified and end-modified through A-tailing and sequence adapter ligating. Finally, by applying PCR based method, the final cDNA library was amplified and subjected to sequencing on Illumina sequencing platform.

Analysis of transcriptome and metabolome was carried out as previous described (Pang et al., 2021; Hong et al., 2022). Metaboanalyst 5.0 was used to process the raw data, and standard deviations were used for data filtering. Data normalization based on sum and automatic data scaling was conducted. Principal component analysis (PCA), one-way analysis of variance, and differential genes screening was performed using Metaboanalyst 5.0, and metabolites with |log2 (FoldChange)| >1 and *P* ≤ 0.05 were designated as differentially expressed in this analysis. To analyze metabolic pathways, the KEGG [Kyoto Encyclopedia of Genes and Genomes (KEGG) and Ortholog database (KO)] (http://www.genome.jp/kegg) website was used to organize all metabolites and pathways information of soybean. In addition, the ClusterProfiler package (v4.6.0) was used to enrich and visualize the KEGG annotation information of the differential accumulated metabolites.

FastQC software (version 0.11.9) was used to qualify the raw RNA-seq reads, and Trim_galore software (version 0.6.7) was used to filter out adapters and low-quality bases. The clean reads were mapped to the soybean reference genome (Glycine_max_v4.0, NCBI) using STAR software (version 2.7.10a). Next, the featureCounts software (version 2.0.1) was used to quantify the subsequent mapped reads. Finally, DESeq2 (version 1.36.0) was used to normalize those quantified reads and analyze differential expression between sample groups treated with saline-alkali and water control. All raw data from this study were submitted to NCBI(BioProject # PRJNA932613). The soybean annotation information was obtained using the R package AnnotationHub (version 3.6.0). Finally, the ClusterProfiler package (v4.6.0) was used to obtain differentially expressed genes (DEGs) to enrich and visualize the analyzed GO and KEGG information.

### 2.8 Combinational analysis of metabolome and transcriptome

#### 2.8.1 KEGG co-enrichment analysis of DEGs and differentially accumulated metabolites (DAMs)

DAMs were simultaneously annotated to multiple metabolic pathways with DEGs to perform KEGG annotation. Following that, the pathways and metabolic pathways enriched in DEGs and DAMs were selected for analysis. ClusterProfiler package (version 4.6.0) was used to filter out the relevant metabolic pathways for enrichment and visualization.

#### 2.8.2 Correlate analysis of DEGs and DAMs

Pearson correlation coefficients (PCCs) between DEGs and DAMs were calculated using the R package WGCNA for the joint analysis. The corresponding *P*-value was performed for screening with the criteria: PCC>0.80 and PCCP<0.05. Pheatmaps plotting was achieved using the R package (version 1.0.12) for the correlation coefficient cluster heat map and Graphics (version 3.6.2) for the nine-quadrant map.

### 2.9 Statistical analysis

SPSS 26.0.0.0 was used for statistical analysis, and GraphPad Prism 8.4.3 was used to visualize the data. Significant differences between means were determined using the least significant difference test (*P* < 0.05) and *t*-test (*P* < 0.05).

## 3 Results

### 3.1 Expression pattern analysis of *AhALDH3H1*

**Figure 1:**
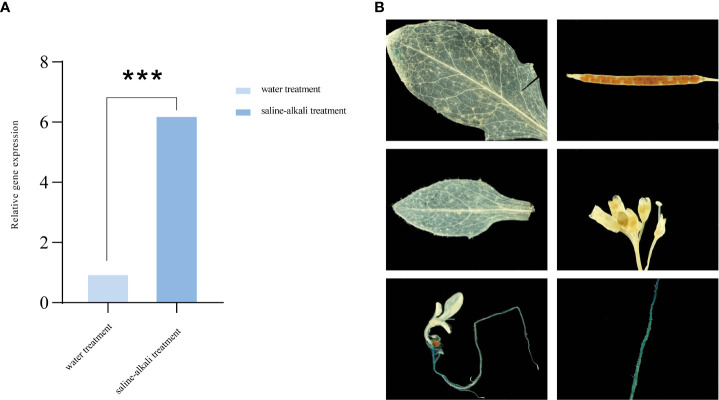
Expression pattern analysis of AhALDH3H1. **(A)** *AhALDH3H1* expression level in *Arachis hypogaea*; **(B)** Tissue-specific activity of the *AhALDH3H1* promoter in transgenic *Arabidopsis thaliana*. Three biological replicates were used. Data was calculated using the 2−ΔΔCT method. Statistical analysis was performed using *t*-test (*P* < 0.05), *P < 0.05, **P < 0.01, ***P < 0.001, and ****P < 0.0001.

*AhALDH3H1* is a previously identified putative gene involved in saline-alkali stress response (Zhang et al., 2023). Therefore, we first evaluated *AhALDH3H1* expression levels in *Arachis hypogaea* at the budding stage under saline-alkali stress and with water treatment as a control. The gene expression increased significantly after 72 h of saline-alkali treatment compared to the water control (**Figure 1A**).

A reporter gene assay is a widely used strategy for gene expression pattern analysis. The temporal and spatial expression of genes are relying on the process that trans-factor recognizes cis-element which located in promoter region and recruits regulating proteins to determine the transcription and translation of target genes. So we can apply promoter of a target gene to drive a reporter gene and then transform into plants, enabling visualization of temporal and spatial expression pattern of the target gene. Here the construct *proAhALDH3::GUS* was developed here to investigate *AhALDH3H1* spatial pattern. The created construct was transformed into *Arabidopsis* ecotype *Col-0* and transgenic lines were screened for expression pattern using spectinomycin antibiotics. *Arabidopsis* organs were analyzed, including rosette and cauline leaves, roots, flowers, siliques, seeds, and young seedlings. *proALDH3H1::GUS* activity was primarily observed in root tissue, pollen tubes, and the distal leaf region. We found significantly higher activity in the early seedling stage, especially in root tissue, which was expected (**Figure 1B**).

### 3.2 Generation of *AhALDH3H1* transformed soybean

To develop transgenic soybean, the construct *pCAMBIA1300-AhALDH3H1* was transformed into a P3 variety. Overall, 16,000 soybean cotyledonary nodes were transformed, and 24 plantlets enabled normal flowering and production of fertile and viable seeds. Based on the LibertyLink test strips testing result for the 24 plantlets, four positive transgenic lines, L1, L2, L3, and L4 were obtained (**Figure S1A**). PCR was then used to confirm whether the target gene *AhALDH3H1* and marker gene *Bar* were present in these four positive transgenic lines. The final amplified fragments were 1473 bp and 403 bp, which matched our expectations (**Figure S1B**). T1 seeds from the four positive transgenic lines were harvested and sown in the greenhouse. Based on the number of harvested seeds, Line L2 was selected for further genetic analysis. We tested *AhALDH3H1* expression level in transgenic soybean under both water-treated and saline-alkali stress conditions. Because *AhALDH3H1* is a saline-alkali induced gene in *Arachis hypogaea*, we investigated whether *AhALDH3H1* has a similar function in soybean. Our findings showed the *AhALDH3H1* gene was significantly expressed under saline-alkali stress conditions (**Figure S1C**), similar to what was observed in *Arachis hypogaea*.

### 3.3 *AhALDH3H1* over-expression increased saline-alkali tolerance of transgenic soybean

**Figure 2:**
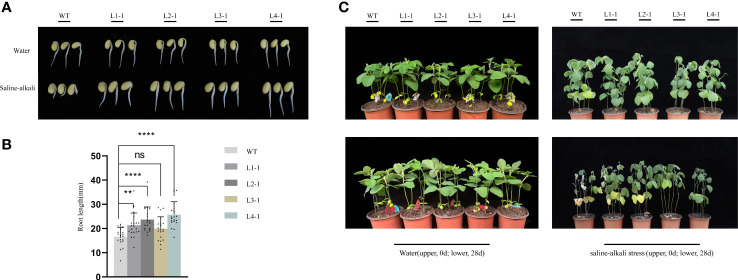
*AhALDH3H1* over-expression increased saline-alkali tolerance of transgenic soybean. **(A)** Germination analysis of *AhALDH3H1* transgenic seeds under saline-alkali stress. Seeds germinated with pH 8.9 saline-alkali solution treatment. WT, wild type. L1-1, L2-1, L3-1, and L4-1, transgenic lines. **(B)** Root length collection of germinated WT and transgenic lines under saline-alkali treatment. **(C)** Morphological changes between transgenic plants and their receptor under water (upper line) and saline-alkali stress (lower line). Statistical analysis was performed using *t*-test (*P* < 0.05), *P < 0.05, **P < 0.01, ***P < 0.001, and ****P < 0.0001. ns, no statistical significance.

We analyzed the function of *AhALDH3H1* based on soybean germination under saline-alkali stress (**Figure 2A**). With water treatment, both WT and transgenic tested seeds germinated normally and displayed a healthy condition. However, with a saline-alkali solution, the germination of WT was inhibited which presented as shorter roots, whereas the tested transgenic lines exhibited normal growth with no difference compared to the seeds germinated without stress treatment. We collected the data of root length and found that, compared to treated with water in which WT and transgenic lines showed no difference in root length, the transgenic lines exhibited a significantly longer root than WT under saline-alkali stress (**Figure 2B**).

To further investigate the effects of *AhALDH3H1* introduction, transgenic lines were subjected to a four-week saline-alkali treatment beginning 14 d post-sowing and we compared the morphological changes after saline-alkali stress. At day 0 of stress treatment, we found no differences between transgenic and WT plants. The plants were then treated with a saline-alkali solution for 28 d, with water treatment serving as a control. Our four transgenic lines exhibited only a slight stress effect and were significantly tolerant to saline-alkali stress compared to WT, which withered and exhibited chlorosis after stress treatment. Specifically, the line L2-1 was outstanding; the plant height, leaf number, and health condition were similar to the plants treated with water and L2-1 did not show a falling leaf phenotype (**Figure 2C**). As a result, we selected this line for further analysis.

### 3.4 *AhALDH3H1* has the characteristics of an aldehyde dehydrogenase

Using a MICHYBIO kit, we measured aldehyde dehydrogenase activity to see if the ALDH3H1 protein had acetaldehyde dehydrogenase activity. The results showed that ALDH activity increased significantly after 14 d saline-alkali treatment in the transgenic line compared to WT (**Figure S2A**). The TBA-based method was used to determine the MDA content. MDA content was significantly decreased in the transgenic line compared to the control plants (**Figure S2B**). The physiological index, which can indicate plant tolerance, was then tested. SOD, a key biological parameter in stress response, is known as an antioxidant enzyme that protects plants from ROS damage. Compared to the water control, SOD activity increased in both transgenic and WT plants treated with saline-alkaline stress. SOD content significantly increased after saline-alkali treatment (**Figure S2C**). These findings indicated that *AhALDH3H1* had aldehyde dehydrogenase activity and that *AhALDH3H1* over-expression in soybean was a key factor in removing MDA produced by saline-alkali stress and increasing SOD content could achieve saline-alkali tolerance.

### 3.5 Transcriptome profiling analysis of *AhALDH3H1* transgenic soybean under saline-alkali stress

**Figure 3:**
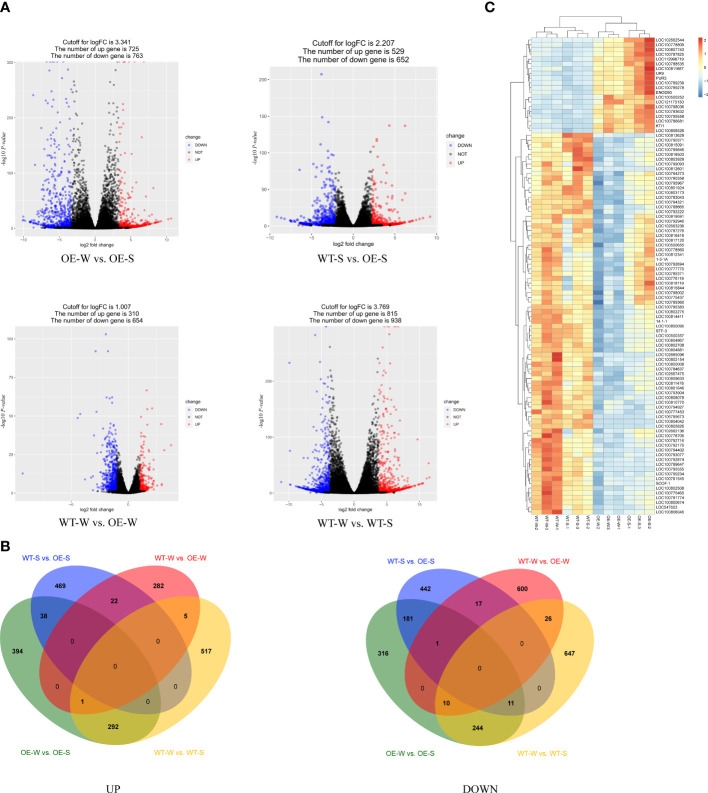
Transcriptome analysis. **(A)** Volcano plot. Red dots represent upregulated differentially expressed genes (DEGs), blue dots represent downregulated DEGs, and black dots represent filtered unigenes that are non-significantly regulated. **(B)** Comparison of DEGs among OE-W vs. OE-S, WT-S vs. OE-S, WT-W vs. OE-W and WT-W vs. WT-S. UP, up-regulated DEGs. DOWN, down-regulated DEGs. **(C)** Cluster analysis of DEGs under saline-alkali stress. The colors from red to blue indicate the highest to lowest log10(FPKM+1) values of each gene under saline-alkali stress or mock water treatment.

In this study, we performed a comparative transcriptome analysis of transgenic soybean-expressed *AhALDH3H1* to determine gene expression profiles under saline-alkali and water conditions. The RNA-seq experiment was designed and conducted with the saline-alkali stress-treated transgenic line L4 and WT root tissues, named OE-S and WT-S, respectively. Similarly, the plants were mock-treated with water and named WT-W and OE-W, respectively. We received 41.73 GB of clean data, with over 3.4 GB of clean data for each sample. We obtained high PCCs based on three biological replicates, indicating that the tested samples had similar expression patterns. We compared the DEGs of four groups in this study: OE-W vs. OE-S (group 1), WT-S vs. OE-S (group 2), WT-W vs. OE-W (group 3), and WT-W vs. WT-S (group 4). The DESeq2 package was used to identify DEGs assigned by those unigenes with an adjusted *P*-value < 0.05. According to the findings, 1488, 1181, 964, and 1753 unigenes were differentially expressed in groups 1, 2, 3, and 4, respectively (**Figure 3A**). For group 1, there were 725 up-regulated DEGs and 763 down-regulated DEGs. For group 2, 529 and 652 up- and down-regulated DEGs were identified, respectively. For group 3, a total of 310 up- and 654 down-regulated DEGs were obtained, respectively. In group 4, there were 815 and 938 DEGs were up- and down-regulated, respectively (**Figure 3B**). To find out the saline-alkali induced genes, we made a comparison among these four groups, we can tell that there were 469 up-regulated and 442 down-regulated DEGs were specific expressed in transgenic lines under saline-alkali stress (**Figure 3B**). Additionally, we compared DEGs between groups 2 and 3 (**Figure 3B**). These two groups showed 22 upregulated genes and 18 downregulated genes. These findings indicate that the introduction of *AhALDH3H1* alters the transcription level of the transgenic soybean and that this type of alteration may contribute to increased saline-alkali tolerance. Next, we specifically compared DEGs in WT and transgenic lines. The down-regulated genes in WT were found to be upregulated in transgenic plants. Further, saline-alkali treatment increased this type of up-regulation (**Figure 3C**). These results suggest that the introduction of *AhALDH3H1* induced the expression of specific genes and saline-alkali stress increased this type of induction, implying that these genes may play a key role in saline-alkali stress response.

### 3.6 Gene ontology enrichment evaluation in *AhALDH3H1* transgenic soybean under saline-alkali stress

**Figure 4:**
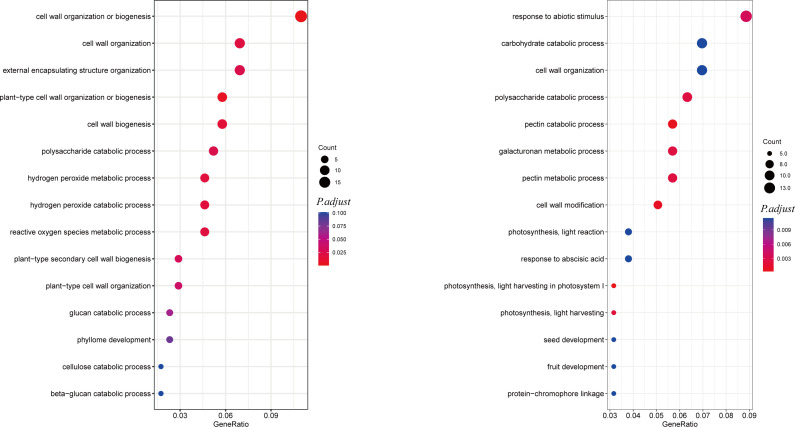
Dotplot of GO enrichment based on up- and down-regulated DEGs from comparing OE-S vs. WT-S. The dotplot GO enrichment of up-(left) and down-(right) regulated DEGs. The y-axis represents GO pathways; the x-axis indicates the gene ratio. The size of the dot is the number of the DEGs that enriched. The color of the dot is the *P*-value, more redder means more significant enrichment.

To better understand the mechanisms by which *AhALDH3H1* responds to saline-alkali stress, we used Gene Ontology (GO) analysis to map the DEGs from RNA-seq data. Removing interference of DEGs that identified from mock-treated group (WT-W vs. OE-W), DEGs from transgenic plants and WT treated with saline-alkali stress (WT-S vs. OE-S) were analyzed. **Figure 4** shows a list of the top 15 most enriched GO terms. This shows that significant GO terms in cell wall organization or biogenesis, cell wall organization, cell wall biogenesis, and plant-type secondary cell wall biogenesis were most enriched for 529 upregulated DEGs. Among the 19 DEGs that were enriched in the GO cell wall organization or biogenesis, 12 of them were critical for cell wall genesis, including three xyloglucan endotransglucosylase genes, one pectinesterase inhibitor gene, five araboinogalactan genes, one pectin acetylesterase gene, and two pectinesterase related genes as well as four expansin genes, all of which were critical for cell wall genesis. In our study, three xyloglucan endotransglucosylase coding genes, including GLYMA_18G165700, GLYMA_13G322500, and GLYMA_03G184500, four expansin coding genes, including GLYMA_18G153500, GLYMA_18G054000, GLYMA_17G260400, and GLYMA_12G111100, and one pectin acetylesterase GLYMA_16G107600 were significantly upregulated in *AhALDH3H1* over-expressed transgenic soybean after saline-alkali stress compared to WT. These findings suggest that the increased tolerance of *AhALDH3H1* transgenic plants to saline-alkali stress is due to increased maintenance of cell wall plasticity mediated by an increased content of xyloglucan endotransglucosylase/hydrolase and expansins, and a thickened cell wall achieved by increasing secondary cell wall strength *via* hemicellulose, such as araboinogalactan and lignin deposition.

The 652 down-regulated DEGs were analyzed to learn how *AhALDH3H1* transgenic soybean responded to saline-alkali stress. Significantly enriched GO terms were identified, including response to abiotic stimulus, polysaccharide catabolic process, pectin catabolic process, and galacturonan metabolic process (**Figure 4**). We reasoned that the disruption in cellular homeostasis caused by saline-alkali stress might compensate for the down-regulation of specific genes involved in catabolic and metabolic processes. Following that, we investigated the significantly down-regulated DEGs that were enriched in the pathways mentioned above. They were found to be more related to chlorophyll a-b binding proteins and pectinesterases. In our study, *chlorophyll a-b binding protein P4* (GLYMA_06G194900), *chlorophyll a-b binding protein 7* (GLYMA_16G016100), and *chlorophyll a-b binding protein 13* (GLYMA_12G219300) were significantly down-regulated in transgenic soybean treated with saline-alkali stress compared to WT, which is consistent with the reported results. Pectinesterase, also known as pectin methylesterases (PMEs), is an important component in cell wall architecture; mediated by PMEs, it affects certain critical cell wall properties that were thought to play a key role in stress adaptation. We found many PME coding genes that were down-regulated after saline-alkali stress treatment. PME41 modulates plant chilling tolerance through a cell wall mechanical properties modification strategy (Qu et al., 2011). PME34 has been reported to have the ability to modify heat tolerance by altering the movement of the stoma (Huang et al., 2017). Pectin was de-esterified into pectate and methanol during pectinesterase catalysis. Therefore, we reasoned that the down-regulation of pectinesterase caused by *AhALDH3H1* transformation resulted in a low content of produced methanol, negatively affecting plant growth. The pressure from saline-alkali stress may be relieved due to the lower methanol level.

### 3.7 Metabolic profiles between water and saline-alkali treated *AhALDH3H1* transgenic soybean

**Figure 5:**
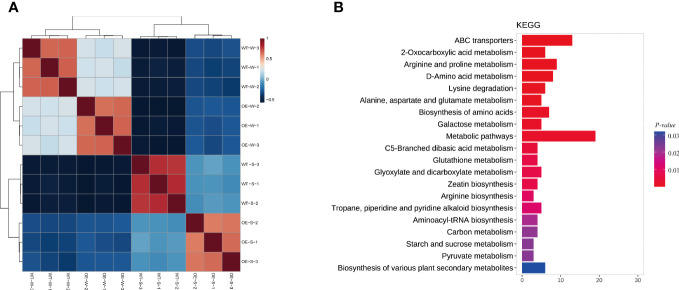
Metabolic profiles between water and saline-alkali treated *AhALDH3H1* transgenic soybean. **(A)** Pearson correlation among QC samples. Red and blue colors indicate high and low correlation, respectively. **(B)** KEGG analysis of significant DAMs. The y-axis represents KEGG pathways; the x-axis indicates gene numbers. Colors from blue to red indicate decreasing *P* value.

A metabolome analysis was performed with soybean root samples based on the UPLC-MS/MS strategy to identify the metabolic profiles of *AhALDH3H1* transgenic soybean under water and saline-alkali conditions. Four groups were distinguished: OE-W vs. OE-S, WT-W vs. WT-S, WT-S vs. OE-S, and WT-W vs. OE-W. We began by examining data repeatability and reliability based on Pearson correlation among quality control samples (**Figure 5A**). For each group, 250, 432, 243, and 89 significant DAMs were identified (**Table 1**). Next, we focused on the group WT-S and OE-S to elucidate the differences in metabolite composition caused by *AhALDH3H1* introduction. We performed a cluster analysis using the obtained DAMs, including 125 up-accumulated and 118 down-accumulated metabolites. Finally, DAMs were used for KEGG analysis to investigate further the pathways obtained by DAMs enriched after saline-alkali stress treatment (**Figure 5B**). These significant DAMs were found to be more enriched in the KEGG pathways, including ABC transporters and metabolic pathways. The ABC transporter is a membrane protein that mediates cellular transport processes. We identified a series of upregulated DEGs related to cell wall genesis in the transcriptome analysis, and the alteration of cell wall architecture caused many metabolite fluctuations. The transgenic plants were able to tolerate saline-alkali stress because of the changing cell wall architecture. Furthermore, over-expression of *AhALDH3H1* induced a decreased level of methanol produced during pectinesterase catalysis. Metabolites, including both necessary ingredients for cell wall genesis and production of harmful substances produced during the saline-alkali stress response, could be transported more efficiently with the help of the ABC transporter. These findings reveal that the over-expression of *AhALDH3H1* elevated the tolerance of transgenic soybean to saline-alkali stress by regulating certain genes related to cell wall architecture and metabolites responsible for various substrates transportation.

**Table 1:** Significant DAMs in different groups.

Group Name	All Significantly DAMs	Down-accumulated	Up-accumulated
OE-W vs. OE-S	250	146	104
WT-W vs. WT-S	432	219	213
WT-S vs. OE-S	243	118	125
WT-W vs. OE-W	89	32	57

### 3.8 Integrated analysis of metabolome and transcriptome

**Figure 6:**
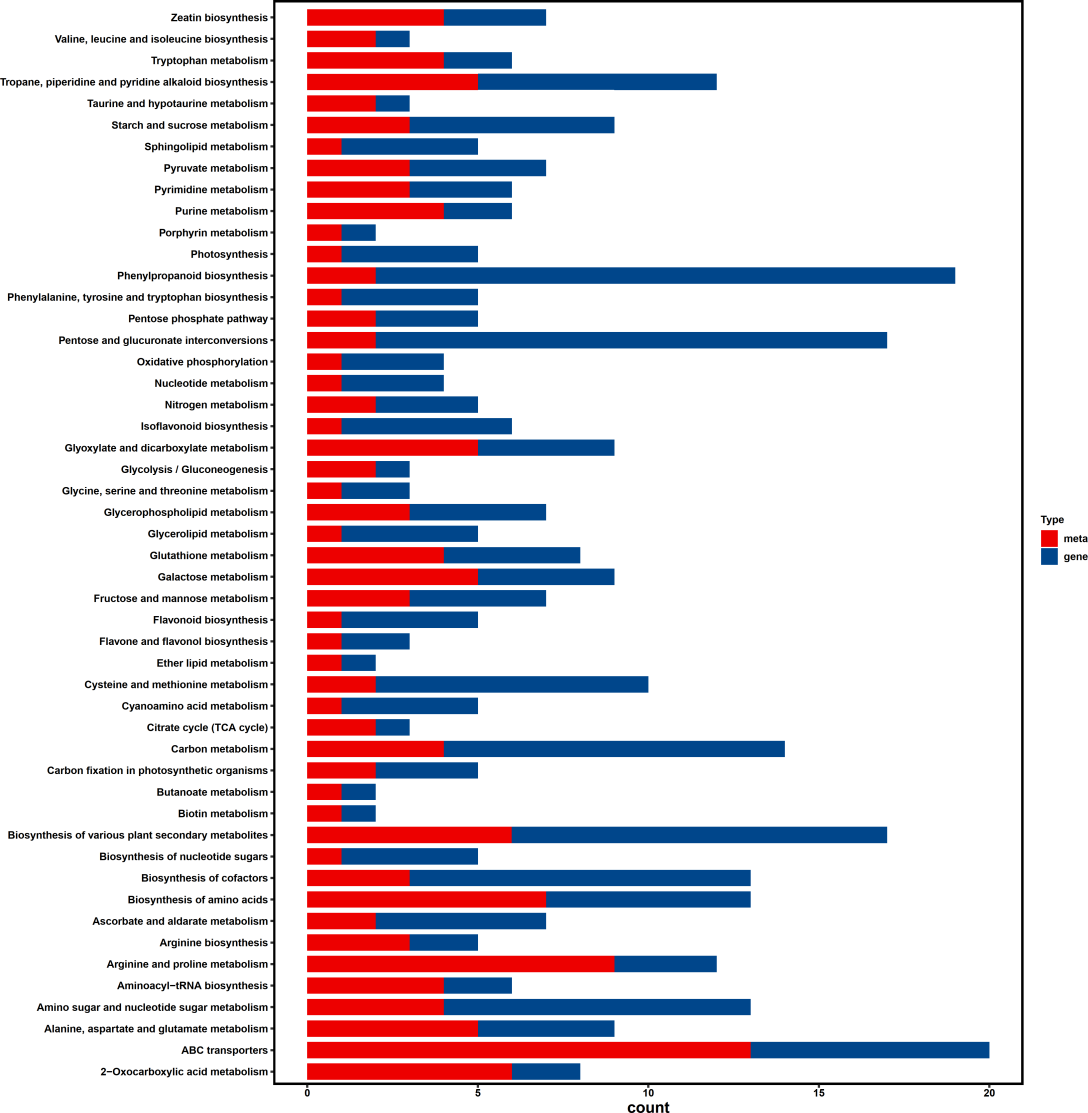
Integrated analysis of transcriptome and metabolome. Different genes and metabolites enrichment bar chart. The y-axis is the number of enriched genes or metabolites. The x-axis represents the enriched pathways. Red color represents the different metabolites, blue colors represents different genes.

**Figure 7:**
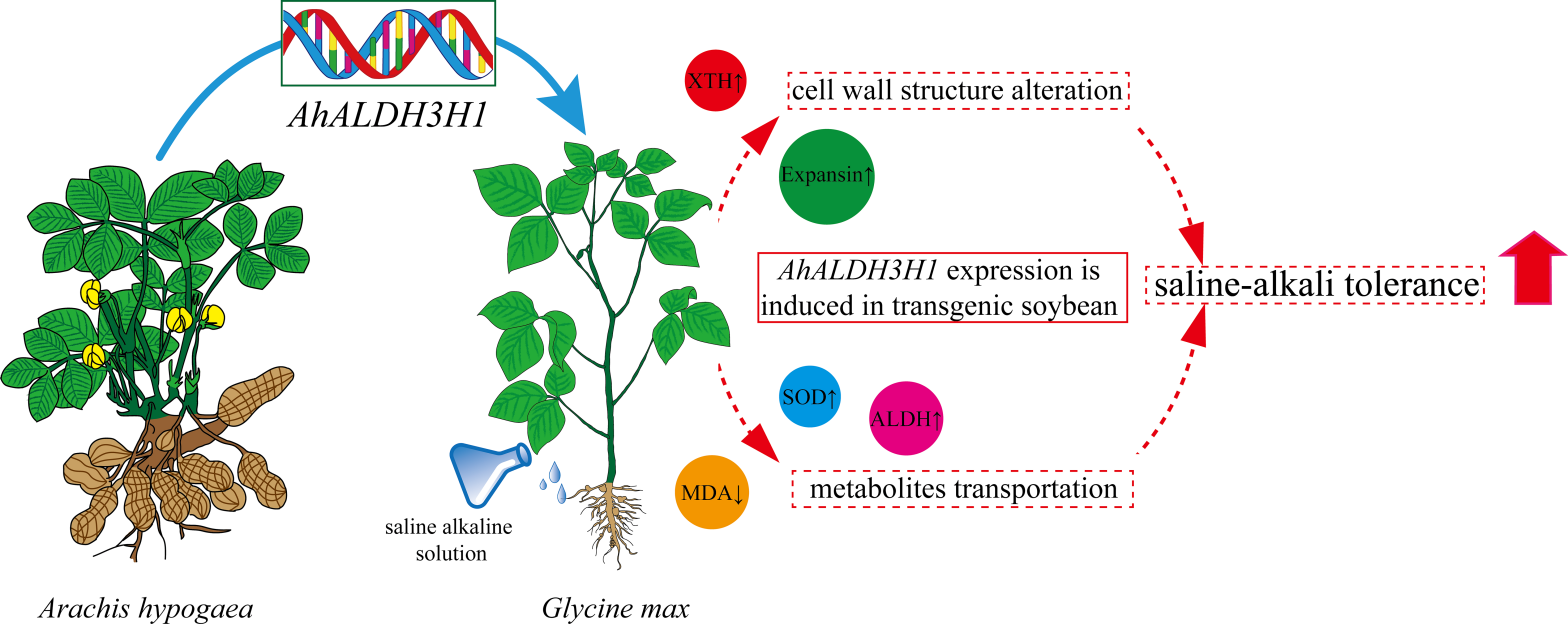
Saline-alkali tolerance regulatory network of *AhALDH3H1* transgenic soybean. The changes of *AhALDH3H1* introduction in soybean under saline-alkali stress were represented as circles which shaded with different colors.

Based on our findings, we conducted metabolome and transcriptome integration analysis to understand the internal mechanisms by which transgenic soybean responds to saline-alkali stress. The results of the association revealed that significant DEGs and DAMs dominated and enriched the biosynthesis of various plant secondary metabolites and ABC transporter pathways (**Figure 6**). In our combined analysis, a series of genes and metabolites involved in cell wall genesis were found to be enriched in secondary metabolites pathways in transgenic plants treated with saline-alkali stress, implying that the introduction of *AhALDH3H1* induced an alteration of the metabolism network under saline-alkali stress. Furthermore, the ABC transporter has been reported to play a pivotal role in mediating polysaccharide transfer to the cell wall and modifying the carbohydrates in the cell wall during abiotic stress. Thus, we mapped the saline-alkali stress tolerance regulatory network of transgenic soybean that expressed *AhALDH3H1* (**Figure 7**). Under saline-alkali stress, the over-expression of *AhALDH3H1* increased soybean tolerance through two different strategies. *AhALDH3H1* over-expression altered cell wall structure by regulating xyloglucan endotransglucosylase related genes and expansin related genes, reducing saline-alkali stress damage. In contrast, produced metabolites like SOD, MDA and ALDH can be transported efficiently by the ABC transporter, which is important in maintaining cell homeostasis inside cells.

## 4 Discussion

Saline-alkali stress significantly impacts soybean growth, development, and reproduction as a major food crop (Wang et al., 2011). Thus, investigating the inner mechanisms of saline-alkaline tolerance, both molecular and physiological, may lead to improvements in saline-alkali tolerance breeding in soybean. Saline-alkali stress is a type of combined stress that is always manifested as a high level of Na^+^ and high pH. Metabolism is disrupted by ion toxicity and further osmotic stress due to the high concentration of Na^+^ in soil (Bhatt et al., 2020). High pH caused by saline-alkali stress significantly impacts pH stability and normal membrane functions. Several mineral nutrients, including Fe, Mg, Zn, and P, will be severely deficient due to high pH (Li et al., 2019). Thus, disruptions in ion balance, osmotic stability, and homeostasis inhibit cell growth and stall plant development, leading to failed crop production (Ganapati et al., 2022). Here, we created an *AhALDH3H1* transgenic soybean more tolerant to saline-alkali stress than WT. The transgenic lines had an obviously healthier phenotype and longer lifespan under stress, indicating that over-expressed *AhALDH3H1* in soybean does increase transgenic line tolerance. The upregulated DEGs between OE-S and WT-S were enriched in the GO terms of cell wall organization or biogenesis, cell wall organization, cell wall biogenesis, and plant-type secondary cell wall biogenesis, based on the transcriptome analysis. The function of the plant cell wall as a protective barrier is related to many proteins. For example, xyloglucan endo-β-transglucosylases/hydrolases and expansins play a critical role in modulating cell wall extensibility which is responsible for cell enlargement and expansion. Further, pectin acetylesterase, which involves the enzymatic deacetylation of pectin, regulates cell wall plasticity/rheology (Le Gall et al., 2015). Plant cell wall structures change in response to abiotic stress. The two main mechanisms involved in plant cell wall stress are widely discussed: one is based on increasing the rhamnogalacturonan I branch that maintains cell wall plasticity by elevating the content of xyloglucan endotransglucosylase/hydrolase and expansins. The other mechanism for increasing cell wall thickness is secondary cell wall reinforcement mediated *via* hemicellulose and lignin deposition. In contrast, the down-regulated DEGs were mainly clustered in response to abiotic stimulus, polysaccharide catabolic processes, pectin catabolic processes, and galacturonan metabolic processes. The plant-specific superfamily of chlorophyll a-b binding proteins is involved in photosynthesis and stress responses. In papaya, 10 d salt treatment led to significantly down-regulated genes, such as *CpLhcb4, CpLhcb1.2, CpLhcb1.3*, and *CpLhca4*. A 15d stress treatment caused an obvious down-regulation of *CpLhcb1.2* and *CpLhcb1.3*, increasing stress duration. These two genes were significantly down-regulated when the treatment was extended to 20 d (Zou et al., 2020). These findings suggest that the downregulation of *CpLhcb1.2* and *CpLhcb1.3* may play a key role in the stress-tolerant process. Following a series of analyses of these enriched DEGs, we concluded that over-expression of *AhALDH3H1* increased transgenic soybean tolerance to saline-alkali stress by increasing cell wall plasticity and thickness was mediated by elevating secondary cell wall strength. Moreover, by regulating photosynthesis efficiency and reducing the level of methanol produced during pectin catalysis, transgenic soybean cellular architecture and homeostasis were maintained in a relatively balanced condition under saline-alkali treatment.

Under saline-alkali stress, ROS, including superoxide and hydroxyl radicals, nitric oxide, singlet oxygen, nitrogen dioxide, and peroxynitrite, are rapidly produced, resulting in lipid peroxidation and gradual alteration of antioxidant enzyme activity (Fu et al., 2017). Several antioxidant enzymes include SOD, peroxidase (POD), catalase (CAT), glutathione peroxidase (GPX), glutathione reductase (GR), and ascorbic acid peroxidase (APX). The antioxidant system has multiple defense lines in plants. The first line is SOD, which can convert superoxide molecules into H_2_O_2_ and oxygen. The following line depends on the enzymes CAT, APX, and POD, which are responsible for converting excess H_2_O_2_ into oxygen and water. MDA is a byproduct of lipid peroxidation, which causes membrane damage. This type of damage can be reduced by removing accumulated MDA when the above-mentioned enzymes are combined. Several genes responsible for saline-alkali stress regulation have been identified over the last few decades (Mitra et al., 2021). For example, the GR family has been identified in several species, including *Arabidopsis thaliana* and *Vigna unguiculata* (Contour-Ansel et al., 2006; Garnik et al., 2016). The APX gene family was identified in *Cucumis sativus*, *Oryza Sativa*, and maize (Jojoa-Cruz et al., 2018). Overexpression of *APX* from *Salicornia brachiata* in peanuts resulted in increased tolerance to salt stress (Singh et al., 2014). Transcription factors have been shown to play an important role in the saline-alkali stress-responsive process. Enhanced salt tolerance can be achieved by overexpression of *SOS1*, *SOS2, SOS3* (Qiu et al., 2002)*, GmSALT3* (Guan et al., 2014), *GmSOS1*, and *GmNHX1* (Zhang et al., 2019; Ma et al., 2020).

ALDH is a detoxifying enzyme family involved in the oxidation of aldehydes to protect cells from the damage caused by reactive and toxic aldehydes. A previous study on potatoes found that by changing the intragenic cytocine methylation status, the expression level of *StALDH2B7a* was upregulated during cold stress and that excess aldehydes produced caused by low-temperature stress can be removed by the ALDH2B7a enzyme (Guo et al., 2020). Moreover, transgenic tobacco with *ScALDH21* overexpression showed increased tolerance to both drought and salt stress (Yang et al., 2015). The *ALDH* family may be used as a candidate in future stress tolerance breeding in plants. Further, ALDH enzymes not only play a key role in aldehyde metabolism, but they play a role in other critical cellular processes such as cell proliferation and differentiation. Several diseases, especially certain cancers, have been linked to ALDH member mutations in previous studies (Ruiu et al., 2019). Thus, the ALDH-based strategy can potentially be a novel cancer treatment method. In this study, we introduced an ALDH coding gene *AhALDH3H1* from *Arachis hypogaea* into the soybean variety P3 genome. We concluded that introducing *AhALDH3H1* into soybean improved transgenic soybean tolerance to saline-alkali stress through cell wall structure maintenance and metabolite transportation. Our findings uncovered a new function of *AhALDH3H1* in the saline-alkali stress tolerance process and may provide a candidate germplasm for future abiotic stress tolerance breeding in soybean.

Saline-alkali stress response is a complex process that requires cooperation of different types of factors and is regulated by multiple mechanisms. In this study, we uncovered the strategies that the transgenic soybean used to enhance saline-alkali stress tolerance and found a series of genes that are responsible for altering cell wall structure and transporting metabolites; however, the specific regulation network requires further study. Other layers of regulation of saline-alkali tolerance like plant hormones should be identified in the future.

## Data availability statement

The data presented in the study are deposited in the NCBI repository, accession number PRJNA932613.

## Author contributions

FM and XZ conceived the research. XZ, LZ, and YC were responsible for experimental design and execution. YC, JW, and SZ performed most of the computational analysis and wet lab experiments. QF and JR performed experiments. SL, YQ, and TL participated in data analysis. YC and FM wrote the manuscript. All authors contributed to the article and approved the submitted version.
