# Supplementary material for: Overexpression of the aldehyde dehydrogenase AhALDH3H1 from Arachis hypogaea in soybean increases saline-alkali stress tolerance
Source: Front Plant Sci. 2023 Mar 28;14:1165384. doi: 10.3389/fpls.2023.1165384 (PMC10086354; doi:10.3389/fpls.2023.1165384)
Supplement: Supplementary file 1 [file DataSheet_1.docx]

Supplementary Material

Overexpression of the aldehyde dehydrogenase AhALDH3H1 from Arachis hypogaea in soybean increases saline-alkali stress tolerance

**Running title**: *AhALDH3H1* increases saline-alkali stress tolerance

**Yingxue Cao^1,2^†, Jing Wang^1^†, Siqi Zhao^1^, Qingxi Fang^1^, Jingwen Ruan^1^, Shuanglin Li^1^, Tongxin Liu^1^, Yuxin Qi^4^, Ling Zhang^*3^, Xiaoming Zhang^*1^, Fanli Meng^*1,2,5^**

^1^Department of Agriculture, Northeast Agricultural University, Harbin, China

^2^Northeast Institute of Geography and Agroecology Chinese Academy of Sciences, Harbin, China

^3^Institute of Agricultural Biotechnology, Jilin Academy of Agricultural Sciences, Changchun, China

^4^Mudanjiang Branch of Heilongjiang Academy of Agricultural Sciences, Mudanjiang, China

^5^Heilongjiang green food science research institute, Harbin, China

*** Correspondence:**

Fanli Meng

[mengfanli@neau.edu.cn](mailto:mengfanli@neau.edu.cn)

Xiaoming Zhang

[xiaomingzhang1982@126.com](mailto:xiaomingzhang1982@126.com)

Ling Zhang

zly_jaas@126.com


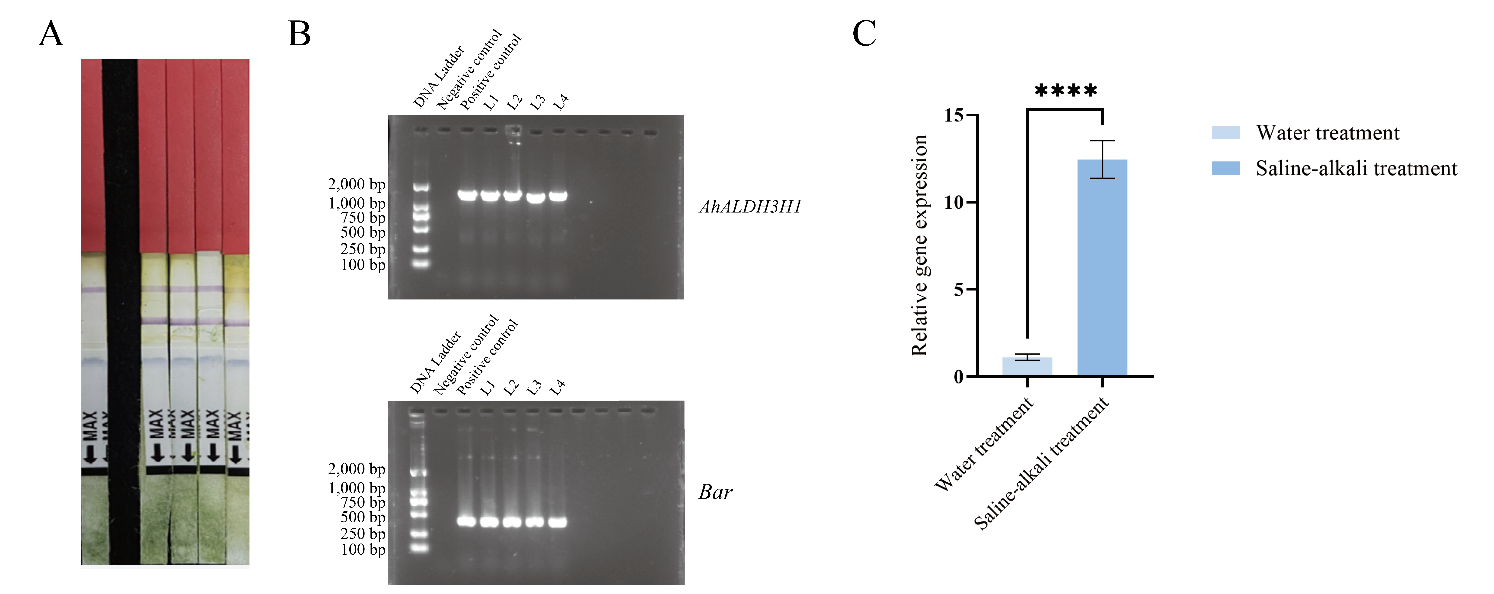


**Supplementary Figure 1.**  **Generation of *AhALDH3H1* transformed soybean and genetic stability analysis. (**A) Bar strip test of transgenic plantlets; (B) Electrophoresis gel figure of PCR test for *Bar* and *AhALDH3H1* in transgenic soybean. (C) Expression level of *AhALDH3H1* in transgenic soybean under saline-alkali treatment. Three biological replicates were used. Data was calculated using the 2^−ΔΔCT^ method. Statistical analysis was performed using *t*-test (*P* < 0.05), **P* < 0.05, ***P* < 0.01, ****P* < 0.001, and *****P* <0.0001. Lane1 to 7 represented DNA ladder, negative control, positive control, transgenic line L1, L2, L3 and L4, respectively.


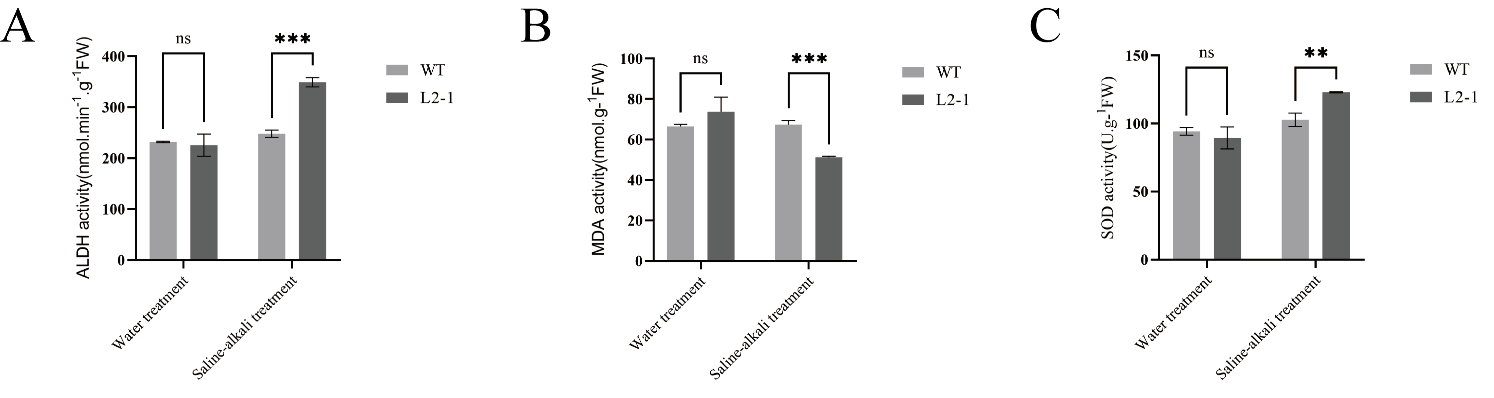


**Supplementary Figure 2.**  **Characteristics of aldehyde dehydrogenase activity of AhALDH3H1**. (A) The activity of aldehyde dehydrogenase of transgenic and wild type (WT) plants under saline-alkali stress; (B) MDA activity of transgenic and WT plants under saline-alkali stress; (C) The activity of SOD of transgenic and WT plants. Three biological replicates were used. Data was calculated as manufacturer’s instructions. Statistical analysis was performed using *t*-test (*P* < 0.05), **P* < 0.05, ***P* < 0.01, ****P* < 0.001, and *****P* <0.0001.

# Supplementary Table1. List of primers used in this study

| Name of primers | Sequences |
| --- | --- |
| AhALDH3H1_qPCR_F1 | GGGAGCTTCCGTTCTGGAAA |
| AhALDH3H1_qPCR_R1 | TCTCGTAGACGACGGTTTCG |
| AhALDH3H1_qPCR_F2 | CGTTGCAGAAGAAGGTTGTGTT |
| AhALDH3H1_qPCR_R2 | CTTCTCTGGCTTCATCCATTGTT |
| AhALDH3H1_test_F1 | GCAGCTGGTAATGCTGTGGT |
| AhALDH3H1_test_R1 | TAACGAGTGCCTTCATCACCC |
| AhALDH3H1_test_F2 | AAGGTTGTGTTTGACGGAGAAG |
| AhALDH3H1_test_R2 | AAAGAGCACCAATGATGCTAAG |
| AhALDH3H1_pro_frg1_F1 | gacctgcaggcatgcaagcttACTGCATATTGTATTATTTTAATATTAAATTACAA |
| AhALDH3H1_pro_frg1_R1 | cgactccatATTCAACGGTCGATCTCTTTTCTT |
| AhALDH3H1_cDNA_frg2_F2 | gaccgttgaatATGGAGTCGTTGCAGAAGAAGG |
| AhALDH3H1_cDNA_frg2_R2 | aatgtcgacggtaccggatccAGCCTTGGAGGACCATCCC |
| AhALDH3H1_pro_2500_F3 | aattaattcctaggagACTGCATATTGTATTATTTTAATATTAAATTACAA |
| AhALDH3H1_pro_2500_R3 | cttcttaggagccataagcttATTCAACGGTCGATCTCTTTTCTT |
| AhALDH3H1_35S_cDNA_F1/2 | attgattagagatcttctagaATGGAGTCGTTGCAGAAGAAGG |
| AhALDH3H1_cDNA_frg2_R2 | aatgtcgacggtaccggatccAGCCTTGGAGGACCATCCC |
| AhALDH3H1_test_F3 | ATGGAGTCGTTGCAGAAGAAGG |
| AhALDH3H1_test_R3 | TTAAGCCTTGGAGGACCATCCCAAA |
